# 2D transition metal carbides (MXenes) in metal and ceramic matrix composites

**DOI:** 10.1186/s40580-021-00266-7

**Published:** 2021-06-02

**Authors:** Brian C. Wyatt, Srinivasa Kartik Nemani, Babak Anasori

**Affiliations:** 1 Department of Mechanical and Energy Engineering, Purdue School of Engineering and Technology, Indiana University-Purdue University Indianapolis, 46202 Indianapolis, IN USA; 2 Integrated Nanosystems Development Institute, Indiana University-Purdue University Indianapolis, IN 46202 Indianapolis, USA

**Keywords:** MXenes, Nanomaterials, Composites, Metals, Ceramics, Mechanical, High-temperature

## Abstract

Two-dimensional transition metal carbides, nitrides, and carbonitrides (known as MXenes) have evolved as competitive materials and fillers for developing composites and hybrids for applications ranging from catalysis, energy storage, selective ion filtration, electromagnetic wave attenuation, and electronic/piezoelectric behavior. MXenes’ incorporation into metal matrix and ceramic matrix composites is a growing field with significant potential due to their impressive mechanical, electrical, and chemical behavior. With about 50 synthesized MXene compositions, the degree of control over their composition and structure paired with their high-temperature stability is unique in the field of 2D materials. As a result, MXenes offer a new avenue for application driven design of functional and structural composites with tailorable mechanical, electrical, and thermochemical properties. In this article, we review recent developments for use of MXenes in metal and ceramic composites and provide an outlook for future research in this field.

## Introduction

**Fig. 1:**
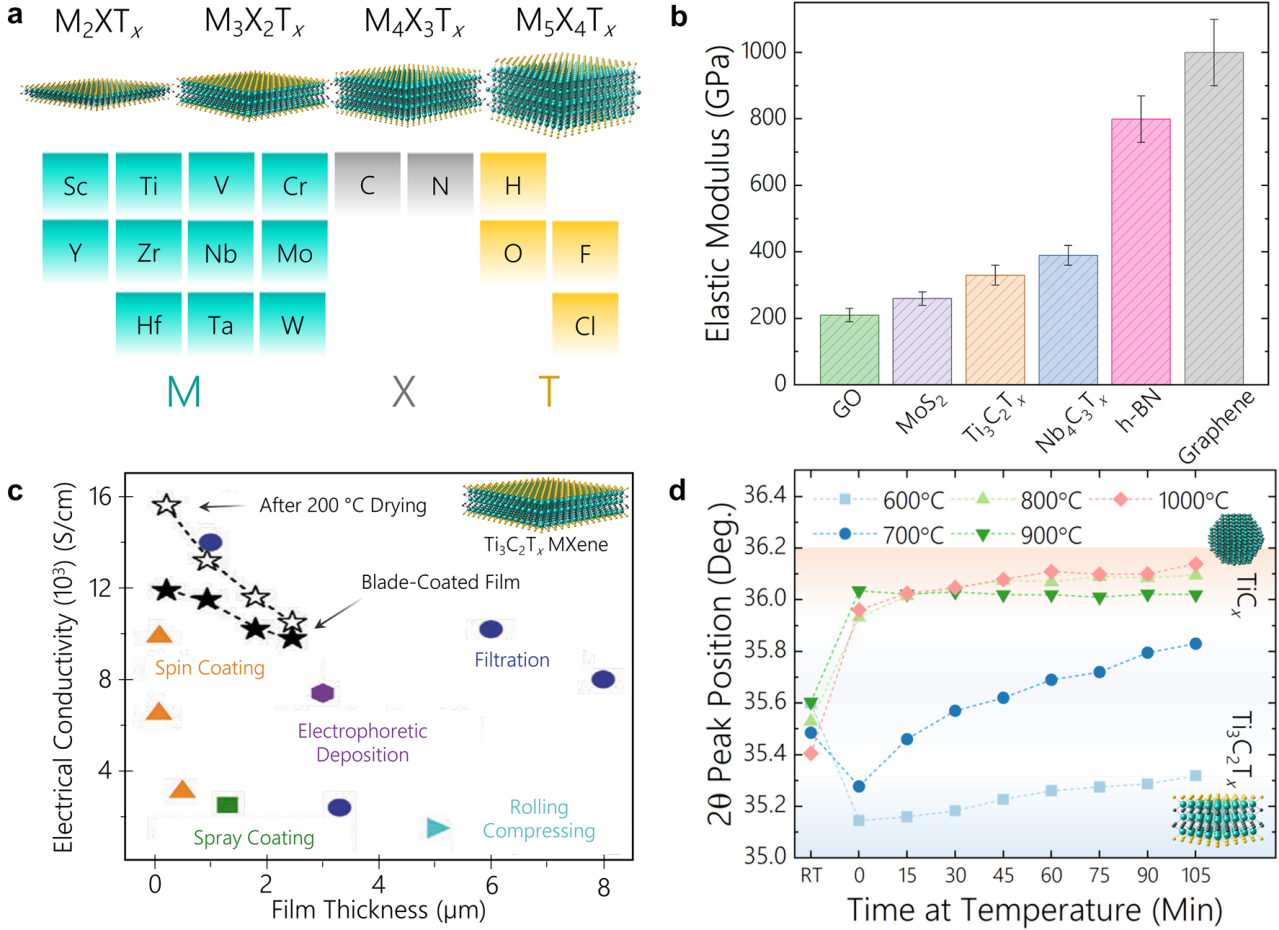
2D MXenes wide array of possible compositions, and impressive mechanical, electrical, and high-temperature properties. **a** MXenes can be comprised of transition metals of groups 3–6 of the periodic table with carbon or nitrogen and are surrounded by mixed surface groups, of which are commonly –O, –F, –(OH), and –Cl. **b** MXenes have shown the highest mechanical stiffness of all solution processable nanomaterials [9, 21]. **c** Ti_3_C_2_T_*x*_ MXenes have shown exceptionally high electrical conductivity depending on the synthesis and fabrication techniques [41]. **d** Ti_3_C_2_T_*x*_ MXenes have shown phase transitions to its highly stable TiC_*y*_ form at temperatures ranging from 700–1000 °C [40]

Two-dimensional (2D) nanomaterials have been under great demand as reinforcing and property tailoring materials in composite applications due to their in-plane mechanical stiffness and strength [1, 2], high chemical activity [3–5], as well as their capacitive and conductive properties [6–8]. 2D MXenes (possible structures of MXenes and their elements are shown in Fig. 1a), have renewed interest to meet this demand due to their impressive mechanical stiffness (up to 386 ± 13 GPa for Nb_4_C_3_T_*x*_) (Fig. 1b) [9], catalytic potential [10], and high in-plane electrical conductivity (up to 20,000 S cm^− 1^ for Ti_3_C_2_T_*x*_) (Fig. 1c) [11]. MXenes are 2D transition metal carbides and nitrides that are denoted by a chemical formula of M_*n*+1_X_*n*_T_*x*_ (*n* = 1 to 4), where M represents 3*d* – 5*d* block transition metals (groups 3–6 of the periodic table) layers which are interleaved by X layers, where X represents carbon or nitrogen [12]. In addition, T_*x*_ represents surface terminations bonded to the outer M layers of MXenes, where T_*x*_ are generally a mixture of –O, –F, –(OH), or –Cl surface groups [12]. MXenes are derived from their carbide precursors (mostly MAX phases). In a MAX phase formula, M and X represent the same elements as MXene while A represents A-group elements, which are commonly from groups 13–16 of the periodic Table [13]. MXenes are synthesized through selective removal of the A-layers in MAX through chemical etching in either aqueous hydrofluoric acid [14–16] or molten salt etchants [17], where the nature and the concentration of the etchants significantly influences the surface functional groups on the resultant MXene structure [18, 19]. The wide array of compositional, structural, and processing choices for MXene permits application-driven design of these nanomaterials to develop a wide array of behaviors [12, 20–22].

MXenes’ impressive properties have led to their inclusion in nanocomposites, which have been implemented in energy-related applications such as supercapacitors [23–25], battery electrodes [26–28], and active catalytic materials [29–31]. In these nanocomposites, MXenes are advantageous additive materials due to a combination of high electrical conductivity, high electrochemical activity, and strong chemical affinity toward oppositely charged species, which assists in active binding [10, 32, 33]. This remarkable behavior of MXenes is also seen in polymer composites, where MXenes add mechanical reinforcement and electrical conductivity while permitting tailorable chemical activity ranging from selective ion sieving, EM wave attenuation, flame retardancy, catalytic activity, and tribological behavior improvement [34].

Similar to these previous successes of MXenes in nanocomposites due to MXenes’ inherent material properties, MXenes have shown potential as functional fillers in metal matrix composites (MMCs) and ceramic matrix composites (CMCs). In addition to MXenes’ noteworthy mechanical and electrical behavior, their high surface charge in a range of solvents [25, 35] permits exploration of solution-based mixing processes. Overall, the strong mechanical properties of MXenes (330 ± 30 GPa and 386 ± 13 GPa for Ti_3_C_2_T_*x*_ and Nb_4_C_3_T_*x*_, respectively) [9, 36] make MXenes the stiffest solution-processable 2D nanomaterials to date (Fig. 1b), which illustrates MXenes’ promise for solution mixing methods. In addition, MXenes inner transition metal carbide/nitride core allows them to be used as a stable high-temperature reinforcement material [37–40]. Although MXenes ~ 1-nm-thick flakes (for M_3_ X_2_T_*x*_ structures) are prone to oxidation in air or aqueous media, MXenes are stable high-temperature phases in dry and oxygen-free environments, such as in encapsulation with metal or ceramic matrices, to meet the greater structural and chemical stability demanded in high-temperature applications.

This high-temperature behavior of MXene flakes in a controlled environment may be categorized in two regimes. First, surface groups are desorbed from the MXenes’ surface in sequential order [37]. For example, Ti_3_C_2_T_*x*_ with T_*x*_ : –O, –F, and –(OH) under high-temperature annealing will lose its surface groups starting with the –OH groups at temperatures of 300–500 °C, followed by –F groups around 500–750 °C, subsequently with complete removal of surface groups above 800 °C [37–39]. Second, annealing temperatures above this point are accompanied by phase transitions of  MXene to TiC_*y*_ superstructures. Previously, we have reported that Ti_3_C_2_T_*x*_ MXene will transform to two forms of 3D crystalline nonstoichiometric TiC at temperatures higher than 700 °C. These phase transformations of Ti_3_C_2_T_*x*_ MXene at temperatures above 700 °C and below 1000 °C result in formation of cubic TiC_*y*_ (0.5 < *y* < 1.0) in an ordered vacancy cubic superstructure of Ti_2_C (only TiC_*y*_ is shown in Fig. 1d for visual simplicity) [40]. At these annealing temperatures, all three phases of Ti_3_C_2_T_*x*_, Ti_2_C, and TiC_*y*_ occupy the layered Ti-C structure simultaneously up to 1000 °C [40]. At temperatures above 1000 °C, the structure transforms to cubic TiC_*y*_ with disordered carbon vacancies. When single-flake Ti_3_C_2_T_*x*_ MXene is used as the starting material, the cubic TiC_*y*_ maintains the layered nature of MXene flakes and transforms in a lamellar morphology [40]. The structural stability of Ti_3_C_2_T_*x*_ MXenes in their hexagonal crystal lattice up to 700 °C and their similar phase integrity for lamellar transition metal carbide/nitride structures at temperatures higher than 1000 °C illustrates MXenes’ promise as a stable 2D nanomaterial and lamellar structures for high-temperature composite materials. The combination of MXenes’ phase stability paired with its aforementioned impressive mechanical and electrical behaviors lend significant promise for MXenes as reinforcement nanomaterials for the next-generation of metal and ceramic matrix composites.

## MXenes metal matrix composites (MMCs)

**Fig. 2:**
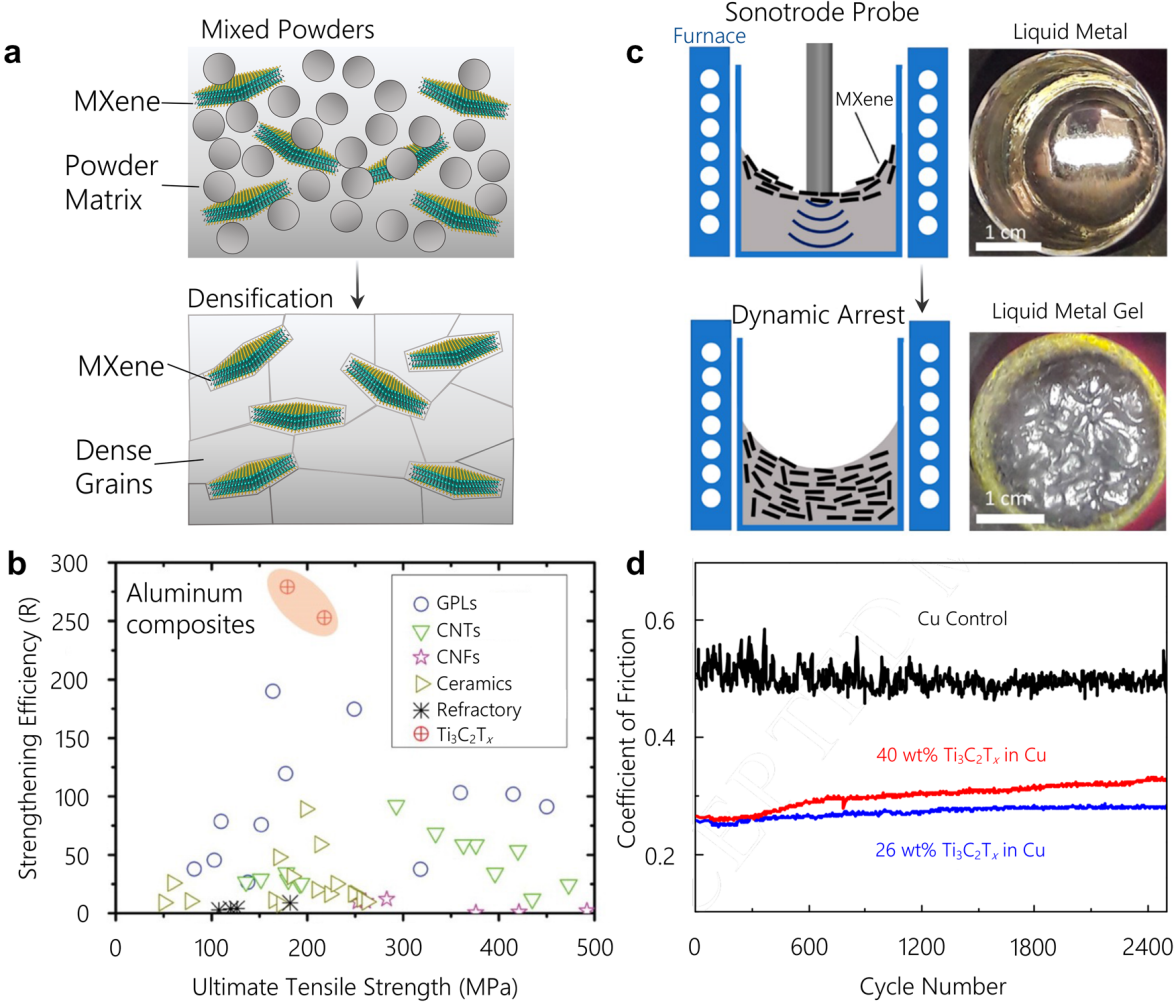
MXenes metal matrix composites, processing and improved mechanical and tribological properties. **a** A schematic representing the MXene single-flake assembly and mixture with metal particles followed by densification to fabricate bulk metal matrix composite. **b** Strengthening efficiency of different nanoparticles and nanoflakes addition into Al matrices showing few-layer delaminated Ti_3_C_2_T_*x*_ flakes can improve the tensile strength over its pure Al matrix with a lower volume fraction than that of other common nano-reinforcement materials [44]. **c** MXenes can also be included into lower melting point metals in their molten phase, where sonication assists the homogenous dispersion of MXene flakes into the molten metal [48]. **d** Ti_3_C_2_T_*x*_ MXene flakes in a Cu matrix have shown a reduction of the coefficient of friction of two times over a pure Cu matrix [49]

MXenes’ strong M-X interior paired with their abundant surface terminations make them a compatible candidate material for use in metal matrix composites for structural and tribological applications. Recent studies have investigated Ti_3_C_2_T_*x*_ MXene as a reinforcement material for Al via powder metallurgy processes [42–44], as shown in Fig. 2a. The first of these mechanical studies on MXene/Al composites mixed non-delaminated multilayer Ti_3_C_2_T_*x*_ powder and Al powder via ball milling followed by pressureless sintering at 650 ºC in an Ar environment, which was further followed by hot rolling [43]. Another study used delaminated few-layer flakes of Ti_3_C_2_T_*x*_ in Al powder mixed in water under sonication, which was followed by spark plasma sintering (SPS) at 580 ºC for 20 min under 50 MPa and further followed by hot extrusion [44]. The use of up to 3 wt% non-delaminated Ti_3_C_2_T_*x*_ powder mixed in Al resulted in a tensile strength improvement of 50 % [42] while the few-layer delaminated Ti_3_C_2_T_*x*_ achieved a tensile strength improvement of 66 % at only 0.2 wt% inclusion of Ti_3_C_2_T_*x*_ in Al [44].

In addition, studies on non-delaminated MXene in Cu matrices for mechanical strength have been investigated. One study utilized an ethanol solution-based mixing method of non-delaminated multilayer Ti_3_C_2_T_*x*_ powder with a Cu precursor using reductive heat treatment at 60 °C to yield Cu followed by SPS at 800 °C under 35 MPa for 5 min with a temperature ramp rate of 50 °C/min [45]. Another study on non-delaminated Ti_3_C_2_T_*x*_ powder in Cu matrices used high-energy ball milling at 350 RPM in Ar to mix the Cu and Ti_3_C_2_T_*x*_ powders followed by vacuum hot pressing at 1040 °C and 25 MPa for 30 min with a temperature ramp rate of 1.5 °C/min to sinter the samples [46]. These studies showed non-delaminated Ti_3_C_2_T_*x*_ powder mechanically reinforced the Cu matrix, with a reported improvement up to 50 % over the pure Cu matrix [45, 46]. While these studies on Al and Cu matrices did not use identical processing conditions, the use of delaminated few-layer Ti_3_C_2_T_*x*_ MXene is expected to be significantly more effective in improving the mechanical properties compare with multilayer Ti_3_C_2_T_*x*_ powder.

In general, the more effective strengthening of single-flake MXene over its multilayer powder counterpart is likely due to the increased surface area for stress transfer from the metal matrix to the delaminated MXene 2D flakes. This increase in surface area has shown to be a very important factor in previous studies of 2D nanomaterial reinforced metal matrix composites [47]. Additionally, the interaction between MXene flakes in a multilayer MXene particle is secondary bonding (e.g., the inter-flake bonding is van der Waals) which is weaker than the primary in-plane M-X bonding. The use of single-flake MXenes will ensure that the MXene materials only have primary bonding as the reinforcing material, which can lead to larger improvements in mechanical properties. The significant improvement in strengthening efficiency of single-flake Ti_3_C_2_T_*x*_ sheets in Al composites as compared to graphene nanoplatelets (GPLs), carbon nanotubes (CNTs), carbon nanofibers (CNFs), and ceramic and refractory particles is shown in Fig. 2b [42]. This graph shows MXene’s potential to improve the mechanical properties of metal composites as compared to competitive reinforcement materials.

Delaminated single-flake Ti_3_C_2_T_*x*_ MXene have also been investigated as an additive material to Mg-Li composites. Ti_3_C_2_T_*x*_ Mg-Li MMCs have been fabricated via a molten-gelation mixing process with Ti_3_C_2_T_*x*_ incorporated into in the molten metal via sonication in temperatures exceeding 500 °C, as shown in Fig. 2c [48]. This process has resulted in a tensile yield strength improvement of 128 % over the matrix metal [48]. Partial phase transformation of Ti_3_C_2_T_*x*_ was noted with a 3 times increase in flake thickness and identification of mixed hexagonal and cubic phases in the recovered flakes [48]. These changes could potentially illustrate partial phase transformations of Ti_3_C_2_T_*x*_ to mixed Ti_2_C and TiC_*y*_ phases as we have observed in high-temperature annealing of Ti_3_C_2_T_*x*_ [40]. The full attribution of this phase transformation in molten phase infiltration [48] can be elucidated with future studies of embedded MXene flakes in MMCs.

The tribological properties of MXene metal matrix composites similarly depends on the mechanical strength and inherent properties of MXenes’ surface terminations. In metal matrix composites under a ball-on-plate setup with a 5 N load, a ~ 2.5 times decrease in the coefficient of friction (COF) of a 3 wt% non-delaminated multilayer Ti_3_C_2_T_*x*_ reinforced Al composite over 300 cycles was attributed to the increased hardness and decreased plastic deformation of the Ti_3_C_2_T_*x*_ reinforced Al composite over pure Al [42]. In a similar configuration under a 1 N load, a 26 wt% delaminated single-flake Ti_3_C_2_T_*x*_ in Cu composite fabricated by electrodeposition processes using multilayer Ti_3_C_2_T_*x*_ and a Cu-containing precursor illustrated 2 times decrease in the COF and a 19 times reduction in the wear rate over its pure Cu counterpart (Fig. 2d). These improvements in wear behavior were attributed to the formed Ti_3_C_2_T_*x*_-tribolayer at the contact point which reduced the required shear force for motion at the interface [49]. The reduced shear force required for contact motion in this tribolayer can be attributed to the low interlayer sliding friction between terminated MXene sheets [50].

We next turn our attention to MXenes unique properties which lend significant promise for their use in metal matrix composites. First, the solution processibility and high negative surface charge of MXene sheets due to its surface groups [25, 35] provides significant potential of MXenes toward solution-based mixing methods, such as electrostatic self-assembly [44]. In addition, MXenes’ status as the stiffest solution-processable 2D nanomaterial makes MXene a great candidate 2D nanomaterial for additive manufacturing and powder metallurgy mixing processes in metal matrix composites for structural applications. Similarly, the surface terminations of MXene provide an anchor point toward chemical bonding of MXene to metal matrices. For example, the effect of –O containing surface terminations on MXenes’ surface has been seen in previous interfacial transmission electron microscopy (TEM) images of few-layer Ti_3_C_2_T_*x*_ to Al, where Al_2_O_3_ forms at the interface between Ti_3_C_2_T_*x*_ and Al [44], which can provide a strong chemical bond for stress transfer during mechanical loading.

Although early studies on MXene metal matrix composites have mostly investigated low-temperature melting metals, which most commonly includes Al and Mg, investigation of MXenes for metal composites with temperatures exceeding 700 °C will certainly have to account for phase transitions of MXenes. Previous studies on the phase transition of Ti_3_C_2_T_*x*_ MXene in a Cu matrix have identified transformation of Ti_3_C_2_T_*x*_ to 3D crystalline TiC_*y*_ phase beginning at 750 °C [46]. This is supported by our previous annealing studies on pure Ti_3_C_2_T_*x*_ films, which identified similar phase transitions that start at 700 °C [40]. In addition, these previous annealing studies on Ti_3_C_2_T_*x*_ illustrate single-flake MXene films’ layered morphology is kept when transformed to 3D crystalline TiC to form nanolamellar TiC_*y*_ grains at temperatures as high as 1500 °C [40]. The stability of Ti-C in metals sintered at temperatures higher than 1000 °C illustrates MXenes’ potential to provide a nanolamellar transition metal carbide reinforcing material for metals with high melting points, which is stable behavior not seen for carbonaceous nanomaterials such as graphene which can turn into undesired phases at higher temperatures [51].

However, the underlying fundamental mechanisms of how MXene mechanically improves the stiffness and strength of metal matrix composites such as surface-group to metal bonding, dislocation motion obstruction, or by-product phase formations has yet to be thoroughly investigated. In addition, studies on the ideal morphology (non-delaminated, few-flake multilayers, or single-flake MXenes) or surface termination compositions for improved MXene metal matrix composite tribological performance may provide further insight into the behavior of MXene composites in low-friction and low-wear applications. Overall, MXene reinforcements in metal matrix composites can utilize MXenes’ mechanically strong interior and conductive behavior in combination with MXenes’ high-temperature stability to form metal composites applied in high-temperature applications. However, the full range of MXenes’ high-temperature stability can be further explored in ceramic matrix composites.

## MXene ceramic matrix composites (CMCs)

MXenes’ high young’s modulus, electrical conductivity, and high-temperature phase stability lend promise for their application in ceramic matrix composites (CMCs). Additionally, MXenes solution processability and high negative zeta potentials (– 32 to – 45 mV) [52] make them great candidates for CMCs’ wet or slurry-based solution processing of green bodies without particle agglomeration as seen in more conventional nano fillers [53]. Also, the negative zeta potential of as-synthesized MXenes eliminates the need for the use of conventional surfactant materials. Solution-processing of MXene CMCs is envisioned to be scalable and can potentially be integrated into existing green body mixing methodologies. MXenes negative zeta potential can be attributed to the presence of surface groups such as –F, –Cl, –O, and –(OH) [54, 55]. These surface charges result in the adsorption of MXene to the oppositely charged ceramic particles due to electrostatic attraction between MXene and the ceramic grains. In addition, MXenes’ extended colloidal stability in non-aqueous solvents such as alcohols as well as processability in dry powder forms can be explored for high-energy mixing strategies. Techniques such as milling, reactive bonding and chemical infiltration may be explored in addition to solution-processing [56, 57]. The effect of dynamic surface chemistries of MXene can also be influential in sol-gel routes for the preparation of green bodies of mixed MXene CMC powders.

**Fig. 3:**
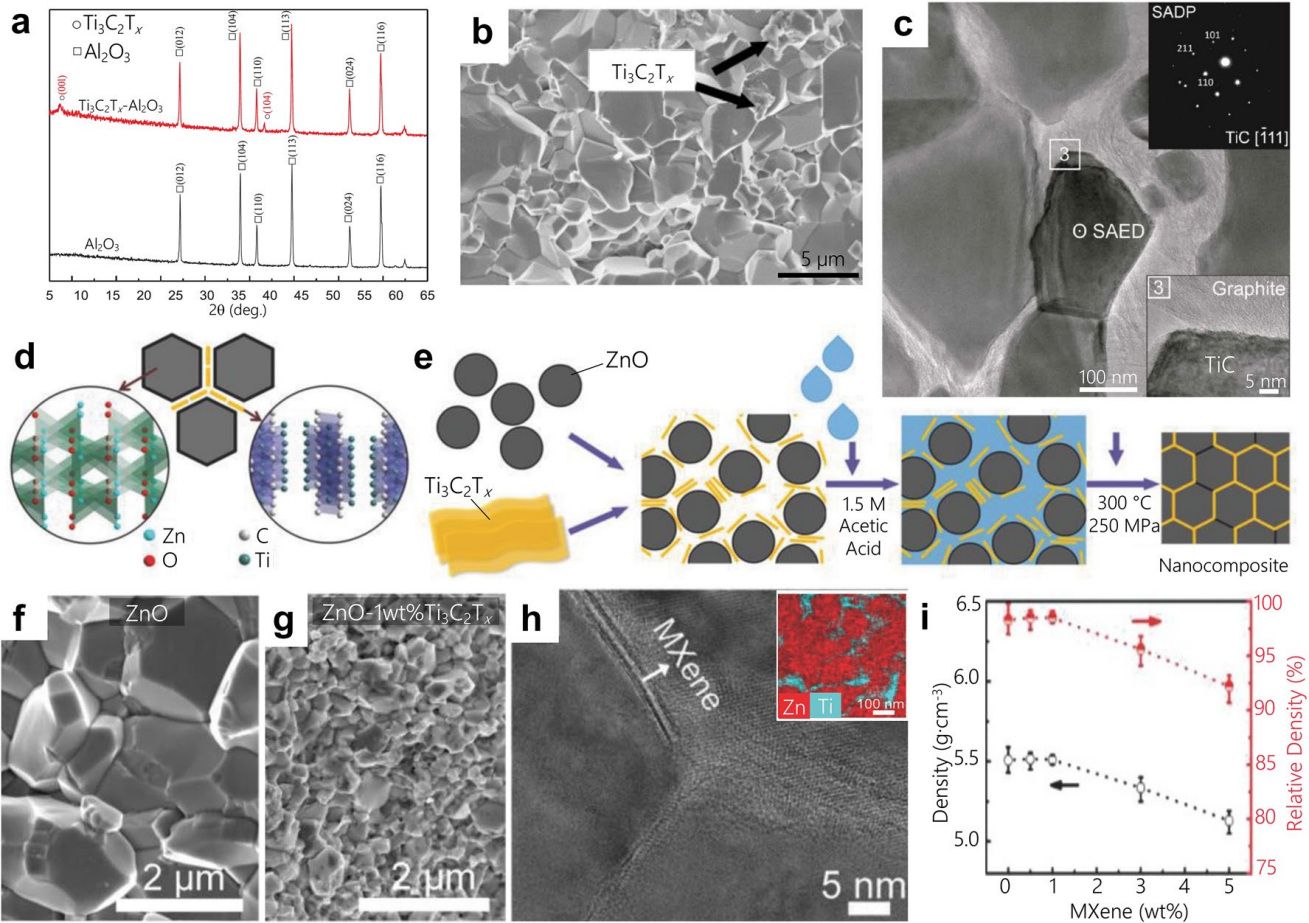
MXenes ceramic matrix composites using matrices such as Al_2_O_3_ and ZnO. **a** XRD patterns of Al_2_O_3_ and Al_2_O_3_—2 wt% Ti_3_C_2_T_*x*_ powder mixtures before sintering [58]. **b** SEM micrographs of Al_2_O_3_—2 wt%Ti_3_C_2_T_*x*_ composite prepared by sintering at 1500 °C [58]. **c** High-resolution TEM image of Al_2_O_3_—2wt% Ti_3_C_2_T_*x*_ with selected area electron diffraction (SAED) of the sintered MXene indicating TiC formation. Bottom inset shows the presence of graphitic carbon at the grain boundaries of the resulting TiC and alumina matrix [59]. **d** Schematic illustration showing (**d**) the grain boundary of ZnO–Ti_3_C_2_T_*x*_ nanocomposites [60] and (**e**) the fabrication process via cold sintering [60]. **f**, **g** SEM images of cols sintered pure ZnO and ZnO—1wt% Ti_3_C_2_T_*x*_ composite [60]. **h** TEM image of a cold sintered ZnO—1wt%Ti_3_C_2_T_*x*_ show the presence of MXene 2D flakes at ZnO grain boundaries. Top inset shows energy dispersive x-ray spectroscopy of ZnO—1wt%Ti_3_C_2_T_*x*_ where Zn is shown in red and Ti is shown in cyan indicating MXenes at the grain boundaries [60]. **i** Densities and relative densities of ZnO–Ti_3_C_2_T_*x*_ nanocomposites cold sintered at 300 °C for 1 h [60]

Preparation of MXene CMC green bodies and sintering routes for MXene-CMCs are dependent on the type of ceramic matrices which can be classified into two categories: oxides and non-oxides. For oxides, alumina and zinc oxide systems have been explored so far. Studies involving alumina as matrix have incorporated only multilayer MXene particles. For example, multilayer particles of Ti_3_C_2_T_*x*_ were mixed with alumina via ball milling in ethanol for 20 h. The resulting green body was sintered in air at 1500 °C for 1 h (Fig. 3a,b) [58]. The composite exhibited an increase in fracture toughness, bending strength, and hardness by ~ 300 %, ~ 150 %, and ~ 300 %, respectively. However, the extended sintering times in an oxygen-rich environment led to MXene phase decomposition to titanium oxide, which can affect the mechanical properties of these composites.

There are methods to mitigate MXene oxidation in ceramic oxide matrices. Recently, sputtered multilayer MXene particles with Mo and Ti have been investigated for incorporation into alumina matrices [59]. Sputtering MXene particles with transition metals was used to create a protective shield to prevent oxidation of MXene in the oxide matrix. The sputtered MXene particles were mixed with alumina (particle size 140 nm) via wet attrition milling in isopropanol. The mixture was sintered via SPS at 1400 °C with uniaxial pressure of 35 MPa and a dwell time of 3 min. The resulting CMCs with 0.5 wt% of Ti_3_C_2_T_*x*_ exhibited an improved hardness (10 %) and fracture toughness (15 %) in comparison to monolithic alumina [59]. High-temperature (1400 ºC) composite sintering turned Ti_3_C_2_T_*x*_ to TiC particles (Fig. 3c) as expected from the high-temperature phase transformation behavior of MXenes. Graphitic carbon was detected at the grain boundaries of these resulting TiC and alumina matrix (Fig. 3c bottom inset). The presence of carbon at the grain boundaries can be related to over etched outer surfaces of Ti_3_C_2_T_*x*_ particles when etched with 48 % hydrofluoric acid for 24 h [38]. However, sintered composites with Mo-sputtered MXene particles show Mo_2_C at the grain boundaries indicating the reaction of Mo with graphitic carbon during the sintering process. Overall, sputtering MXenes with transition metals was shown to be an effective method to improve the oxidation resistance of MXenes specially when incorporated in oxygen-rich matrices and improve the mechanical properties [59]. Further studies are needed to fully understand the effects of the metallic sputtering at the interfacial regimes between metal-MXene, metal-oxide matrices and MXene-oxide matrices.

Another method to prevent MXenes’ oxidation when mixed with oxide matrices is low-temperature sintering processes. Cold-sintering has been explored for single-flake Ti_3_C_2_T_*x*_ MXene mixture with submicron ZnO particles. The composite mixtures were prepared by mixing ZnO particles with 0.5 to 5 wt% of Ti_3_C_2_T_*x*_ single-flake solution and sonicated for 15 min, followed by freeze-drying for 72 h. The resulting ZnO-Ti_3_C_2_T_*x*_ powders were mixed with ~ 20 wt% of 1.5 M acetic acid (1 g of powder with 0.2 g of acetic acid), and the wet powders were sintered at 300 ºC for 1 h at 250 MPa [60]. The schematic of cold sintering method of ZnO-Ti_3_C_2_T_*x*_ composites is shown in Fig. 3d, e. Addition of MXene flakes at the grain boundaries prevented ZnO grain growth (compare Fig. 3f and g). MXenes also enhanced the electrical conductivity of ZnO by 5 orders of magnitude with the addition of 5 wt% Ti_3_C_2_T_*x*_. Figure 3h shows the presence of MXene flakes at the grain boundaries of ZnO, which provided an efficient pathway for the electron transport to improve the electrical conductivity of ZnO–Ti_3_C_2_T_*x*_ CMC [60].

In non-oxide ceramics, CMCs have also taken advantage of MXenes’ strong mechanical stiffness, 2D layered structure, as well as the electromagnetic interference (EMI) shielding properties. In a study, Ti_3_C_2_T_*x*_ multi-layer particles with Si_3_N_4_ were ball-milled in isopropanol media for 10 h [61]. The green bodies were sintered via SPS at 1750 ºC with a dwell time of 30 min and a uniaxial pressure of 30 MPa. The characterization results indicated that addition of an oxide sintering additive, such as ZrO_2_, was needed to achieve high densification. However, when the oxide additive was used, the sintered composite showed no indication of MXene in the final CMCs. Despite MXene transformation to oxide and formation of Si_2_N_2_O, the addition of Ti_3_C_2_T_*x*_ enabled the modification of the phase composition of the ceramic by limiting the α-Si_3_N_4_ → β-Si_3_N_4_ phase transition [61]. At 5.2 MPa m^1/2^, the fracture toughness of the 0.7 wt% Ti_3_C_2_T_*x*_-ZrO_2_-Si_3_N_4_ composite was measured to be 15 % higher than that of a pure Si_3_N_4_ sintered at similar conditions. In another study, Ti_3_C_2_T_*x*_ MXene was mixed with a polymer (hyperbranched polyborosilazane) at 3–10 wt% and after polymerization at 400 °C for 2 h followed by pyrolyzation at 1000 °C and subsequent annealing, a TiC/SiBCN ceramics were fabricated. The resulting MXene-derived TiC reinforced SiBCN composite exhibited good absorption in the X-band as well as a stable performance at higher temperatures (up to 600 °C) in argon as well as air atmospheres [62]. Overall, the layered formation of nanocrystalline non-stoichiometric TiC_*y*_ crystals at the grain boundaries is likely the reason for the CMCs’ higher thermal stability.

Ti_2_CT_*x*_ MXene was used as a filler to develop SiC composites [63]. 1 to 3 wt % Ti_2_CT_*x*_ few-layer MXene flakes were solution-processed in isopropanol and mixed with β-SiC (particle size 420 nm) in a planetary ball mill for 10 h. The green bodies were then sintered via SPS in a vacuum at 1900 °C for 30 min with a heating rate of 50 °C/min and applied uniaxial pressures of 50 MPa. Sintered CMCs exhibited improved densification with relative densities of 99.5 % in composites with 1 wt% Ti_2_CT_*x*_ loading when compared to pure SiC (~ 98.5 %). A nominal 10 % increase in hardness and a 66 % increase in fracture toughness were also reported for composites with 1 wt% MXene content against pure SiC. MXenes’ behavior as a sintering aid for obtaining densified SiC composites can further be expanded to study other carbide systems and their behavior with MXene as fillers.

MXene ceramic composites are in their infancy and there are limited number of studies in this field. However, since MXenes transform to bulk carbides at high temperatures [40], the wealth of knowledge on bulk 3D crystalline carbides as reinforcements in CMCs can help our understanding and expectations of MXene CMCs. In general, the addition of secondary fillers is shown to improve densification, sinterability, and enhanced oxidation stability, while preventing excessive grain growth in CMCs [64–67]. Monocarbide 3D crystalline filler phases such as SiC, WC, VC are conventionally used to improve the processing of high-temperature materials such as ZrB_2_, HfB_2_ ceramics [68–70]. The presence of transition metal carbide phases at the grain boundaries of the ceramic matrix has been demonstrated to improve the bending strength of the composite materials by improving intergranular bonding between ceramic grains through diffusion [71]. This intergranular bonding is due to inherent synergetic phase compatibility between carbide reinforcements and high-temperature ceramics [72]. In addition to intergranular strengthening, these carbide phases can also provide a range of advantageous material properties to CMCs due to their intrinsic mechanical and electrical properties while also improving their oxidation stabilities [73–75]. MXenes are a particularly promising carbide nano filler, as they can be considered ordered carbon vacancy carbides [40] with strong interior M-X ionic/covalent bonding capable of forming primary bonds with ceramic matrices due to their surface groups.

## Future outlook of MXene as a high-temperature additive

MXenes’ inherent strength and conductivity paired with their compatibility toward solution-processibility in mixing with metal and ceramic matrices makes them a particularly strong candidate for future metal and ceramic matrix composites. In general, MXenes’ status as a highly stiff solution-processable 2D nanomaterial with abundant surface groups makes MXene a strong candidate as compared to other nanomaterials that require structurally detrimental processes of mixing and densification, or additives to create surface functionalities capable of similar behavior as MXenes.

Future studies on structural MXene metal matrix composites should take advantage of single-flake MXenes for their high surface area and available surface terminations for strong interfacial bonding for stress transfer. Furthermore, recent studies toward surface group functionalization [18] illustrate potential for tuning MXenes surface group to mediate chemical bonding of MXenes to the metal matrix, which can provide a strong interface for stress transfer in structural metal composites. In structural applications requiring the use of multilayer flakes of MXenes, studies on the wettability of MXenes with the metal matrix will be necessary to ensure the metal matrix can be partially infiltrated into the layers of MXene flakes to prevent detrimental van-der-Waals sliding interactions of the 2D flakes in the MXene particles.

**Fig. 4:**
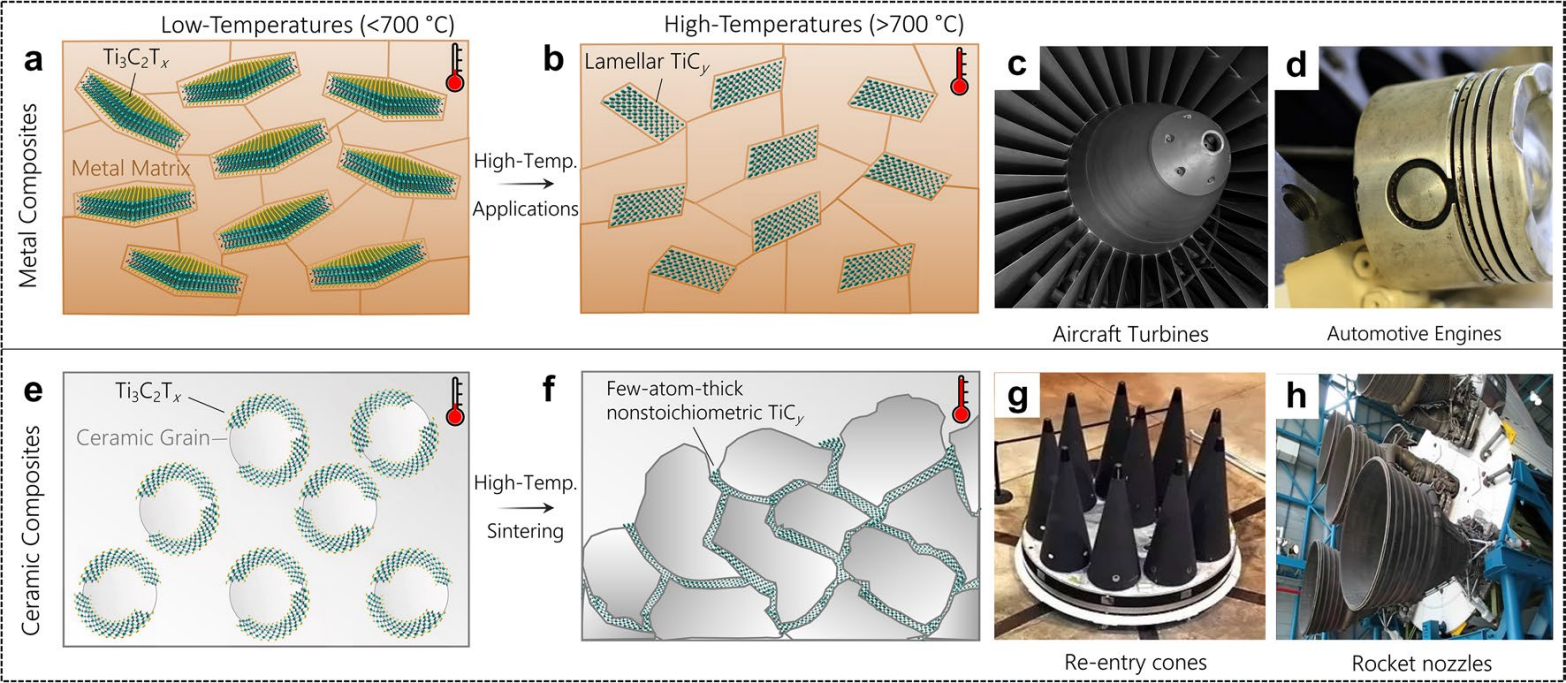
Future applications of MXene metal and ceramic composites. **a**, **b** MXenes can be utilized as reinforcement materials in single-flake form (**a**) or be converted to lamellar TiC_*y*_ in metals under high-temperature applications (**b**). **c**, **d** Some of these high-temperature applications may require strong mechanical properties such as aircraft turbines (**c**) or low friction applications such as automotive engine cylinders (**d**). **e**, f MXenes’ 2D nature can be utilized to wrap ceramic grains (**e**) to undergo phase transformation to strong and conductive chemically bonded intergranular reinforcement materials (**f**). **g**, **h** The application of MXenes toward strong-intergranular bonding can provide use for MXenes as ultra-high temperature ceramic materials for aerospace re-entry cones (**g**) or rocket nozzles (**h**). In panels **a**, **b**, **e**, and **f** schematics, the size of MXene flakes versus the matrix grains are not proportional for the sake of visual clarity

In current studies on MXene metal matrix composites, their mechanical and tribological enhancement behavior thus far have indicated MXenes’ ability to perform as stable additives, as shown in Fig. 4a. This impressive behavior of MXenes in low-temperature metals thus far paired with their high-temperature stability indicates their potential for high melting-point metals, as shown in Fig. 4b. The formation of lamellar carbides within high-melting point metals could potentially reap benefits from interfacial bonding between the M-C structure and the metal mediated by surface groups, which could result in improved mechanical and tribological properties of the composite material. In future application studies of MXene metal matrix composites, additive manufacturing routes such as laser sintering, which is already used for other 2D materials [76, 77], can be adopted to create dense metal composites from powder mixtures of MXene and their metal matrices. MXenes own a particular promise for additive manufacturing of metal matrix composites due to their inherent solution processability and negative surface charge, which can potentially be used to create scalable solution mixed batch sizes for industry-level additive sintering processes. Future studies on this topic should analyze the resultant mechanical properties and phase stability of MXenes during the additive manufacturing processes. These high-melting-point metal-MXene composites could see use in applications where rapid thermal fluctuations are a concern to the mechanical integrity, such as aircraft turbines shown in Fig. 4c, or where low-friction is a concern, such as engine pistons as shown in Fig. 4d. However, future studies of phase transformations and reactions of MXene with metals will be necessary to characterize the phase transformations within the metal matrices.

In ceramic composites, the highly stiff and electrically conductive M-X core of MXenes lends promise for MXenes to be used as additives to improve fracture properties and electron transport in CMCs. In addition, the large amount of possible transition metal carbide or nitride combinations of MXene paired with MXenes’ solution processibility illustrates MXenes’ potential to be compositionally tailored for improved grain-boundary bonding in structural ceramic matrix composites. Self-assembly processes can be implemented to design and develop single MXene flake-wrapped ceramic grains with extremely low volumetric loadings of the filler, that is MXene, in the CMCs, as shown in Fig. 4e. MXenes stability and phase transformation to lamellar carbides in oxygen-free environment [40], such as boride, carbide, and nitride matrices make MXenes the only high-temperature carbide 2D nanomaterials. The high-temperature stability of MXenes can be used to develop CMCs with a greater degree of grain refinement and crack arrest behavior due to a conformal presence of MXene flakes on the ceramic matrix grains, as shown in Fig. 4f. The fundamental methods of how MXene behaves in ceramic matrix composites such as interfacial bonding, crack blunting, or synergetic chemical behavior have yet to be thoroughly investigated. Similarly, a full characterization of phase transformations at the interface of MXene to the ceramic grains during sintering has yet to be conducted.

Future prospects in the design of MXene incorporated CMCs are very promising, with many exciting properties yet to be explored and evaluated. In addition to improvement in conventional mechanical properties of CMCs, MXenes have the potential to enhance electrical, optical, and magnetic properties of materials for applications such as EMI shielding, capacitive, dielectric, and electrically conductive additives, or energy storage. MXenes may be explored as fillers in additive manufacturing strategies for CMCs. Their high solution compatibility and ability to disperse without forming agglomerates may be harnessed in developing 3D-printing strategies for micro and macrostructural engineering. Beyond regular ceramic composites, MXene’s transformation to lamellar carbides opens their applications in ultra-high temperature ceramics (UHTCs) in fields such as space explorations extra-terrestrial landings, and space science such as re-entry cones (Fig. 4g) or rocket nozzles (Fig. 4h). Future studies will need to investigate the mechanical behavior such as fracture, thermal stresses, and fatigue behavior in cyclical loadings of both non-phase transformed and phase transformed MXenes flakes at the UHTC grains to identify the ideal MXene compositions and structures for these applications.

## Conclusions

Since their discovery in 2011, the impressive behavior of MXenes has led to their application in a large range of applications. Although recent studies have started to investigate MXene as a reinforcement material in metal and ceramic matrix composites, there still remain many possibilities for MXenes to revolutionize these composite materials which have yet to be experimentally explored. The mechanical, tribological, and electrically conductive behavior of MXenes paired with their large range of transition metal, carbon or nitrogen, and surface group compositions lends significant potential for application-based design of MXenes for metal and ceramic matrix composite applications. In addition, MXenes’ highly stable transition metal carbide and nitride core at temperatures exceeding 1000 °C means that MXene is potentially the most promising reinforcing 2D nanomaterial for high-temperature applications of metal and ceramic matrix composites. We believe that the impressive potential of MXenes will result in MXenes becoming the leading nanomaterial reinforcement in composites to meet the high-temperature applications of tomorrow’s metal and ceramic matrix composite materials.

## Data Availability

Reference to the datasets analyzed during the current study are available from the corresponding author on reasonable request.
